# Pervasive Sign Epistasis between Conjugative Plasmids and Drug-Resistance Chromosomal Mutations

**DOI:** 10.1371/journal.pgen.1002181

**Published:** 2011-07-28

**Authors:** Rui F. Silva, Sílvia C. M. Mendonça, Luís M. Carvalho, Ana M. Reis, Isabel Gordo, Sandra Trindade, Francisco Dionisio

**Affiliations:** 1 Centro de Biologia Ambiental, Faculdade de Ciências da Universidade de Lisboa, Lisboa, Portugal; 2 Instituto Gulbenkian de Ciência, Oeiras, Portugal; 3 Departamento de Biologia Vegetal, Faculdade de Ciências da Universidade de Lisboa, Lisboa, Portugal; 4 Center for Biodiversity, Functional, and Integrative Genomics (BioFIG), Faculdade de Ciências da Universidade de Lisboa, Lisboa, Portugal; Agency for Science, Technology, and Research, Singapore

## Abstract

Multidrug-resistant bacteria arise mostly by the accumulation of plasmids and chromosomal mutations. Typically, these resistant determinants are costly to the bacterial cell. Yet, recently, it has been found that, in *Escherichia coli* bacterial cells, a mutation conferring resistance to an antibiotic can be advantageous to the bacterial cell if another antibiotic-resistance mutation is already present, a phenomenon called sign epistasis. Here we study the interaction between antibiotic-resistance chromosomal mutations and conjugative (i.e., self-transmissible) plasmids and find many cases of sign epistasis (40%)—including one of reciprocal sign epistasis where the strain carrying both resistance determinants is fitter than the two strains carrying only one of the determinants. This implies that the acquisition of an additional resistance plasmid or of a resistance mutation often increases the fitness of a bacterial strain already resistant to antibiotics. We further show that there is an overall antagonistic interaction between mutations and plasmids (52%). These results further complicate expectations of resistance reversal by interdiction of antibiotic use.

## Author Summary

Bacteria can become resistant to antibiotics by spontaneous mutation of chromosomal genes or through the acquisition of horizontally mobile genetic elements, mainly conjugative plasmids. Plasmid-borne resistance is widespread among bacterial pathogens. Plasmids generally entail a cost to the host, associated with the replication and maintenance of the genetic element and with the expression of its genes. Therefore, in the absence of antibiotic, both plasmids and resistance mutations are often deleterious and confer a fitness cost to the cell. Here we studied epistatic interactions between five natural conjugative plasmids and ten chromosomal mutations conferring resistance to three types of antibiotics, making a total of 50 different combinations of chromosomal mutations and conjugative plasmids. We show that sometimes plasmids confer an advantage to bacterial strains carrying resistance mutations in their chromosome. This occurs in 32% (16 out of 50) of tested combinations. Furthermore, in 5 out of 50 plasmid-mutations combinations studied (10%), we observed an increased fitness when a plasmid-bearing bacterial cell acquires a drug-resistant mutation. These examples of sign epistasis are highly unexpected. This work explains, at least in part, how multidrug resistance evolved so rapidly.

## Introduction

Multidrug resistance is a major hurdle for modern medicine, putting at risk commonplace medical practices [1] and the treatment of infection by bacterial pathogens [2]. Bacteria can become resistant by spontaneous mutation of chromosomal genes or through the acquisition of horizontally mobile genetic elements [1]. In the absence of antibiotics, resistance mutations are often deleterious and confer a fitness cost to the cell [3], [4], [5], [6]. It is logical to expect that, in the absence of antibiotic selective pressure, resistant strains will be outcompeted by the susceptible ones. Thus, a possible procedure to eliminate resistance is to ban the use of an antibiotic. This policy has been applied in different countries with varying results. For example, a deliberate reduction in the prescription of macrolides in Finland, resulted in a 50% decrease in the frequency of macrolide-resistant group A streptococci [7]. However, in the UK, a 98% decrease in the consumption of sulfonamides was accompanied by an increase of 6.2% in the frequency of sulfonamide resistance in *Escherichia coli* [8]. Clearly, there are other factors affecting the reversal to susceptibility. For example, resistant-bacteria often gain second-site mutations that ameliorate the fitness cost of resistance [3], [4], [9], [10], [11], [12], [13]. Sometimes, compensatory mutations even increase the level of resistance itself [14], [15].

The exchange of accessory genetic elements, in particular of conjugative plasmids, is frequent [16], [17], [18] and can disseminate genes among related and phylogenetically distant bacteria [16], [18], [19]. In addition, conjugative plasmids are able to mobilize other plasmids from a donor to a recipient cell [20]. Thus, resistance genes can quickly spread among bacterial communities. Plasmid-encoded resistance is generally the result of the activity of efflux pumps, agent-modifying enzymes [3], or protection of the antibiotic target [21].

Harboring mobile genetic elements generally creates a cost to the host, associated with the replication and maintenance of the genetic element and with the expression of its genes. Such cost has been experimentally demonstrated in a number of resistance-encoding plasmids [22], [23], [24], [25], [26].

A recent study of the interaction between resistance-determining chromosomal mutations, responsible for resistance to nalidixic acid, rifampicin and streptomycin in *E. coli*, found that, in the majority of the cases, the combined fitness cost of double resistance is smaller than one would expect if they were independent [27]. Gene interaction, or epistasis, is generally accepted as being relevant for the understanding of the evolution and dynamics of complex genetic systems [28]. Epistasis can vary in strength and form. When epistasis affects fitness, one can expect two possible outcomes. A positive epistatic interaction has an antagonistic effect on deleterious mutations. Thus, the double mutant has a higher fitness than the expected sum of costs. Negative epistasis between deleterious mutations creates a synergistic effect. Here, the double mutant is less fit than the expected sum of costs. Different studies of epistasis gathered evidence for both antagonistic and synergistic gene interaction. Positive epistasis between random deleterious mutations has been experimentally detected in phage ΦX174 [29], HIV-1 [30], RNA virus Φ6 [31], *Salmonella typhimurium* [32] and in the yeast *Saccharomyces cerevisiae* [33]. Other studies have found no evidence of epistatic interactions within the HIV-1 transcriptional promoter [34], in RNA virus [35], [36] and in *S. cerevisiae* [33], or evidence that positive epistasis occurs as often as negative epistasis in RNA virus [37] and in *E. coli* [38].

Here we focus on the interplay between conjugative plasmids and chromosomal mutations in *E. coli*. In particular, we look at how bacterial fitness is affected by genetic interactions between plasmids and resistance mutations. First, we quantify the degree of epistasis between five conjugative resistance plasmids (R124, R831, R16, R702 and RP4, carrying between one and four resistance genes and belonging to four different incompatibility groups) and 10 mutant alleles of the housekeeping genes *gyrA*, *rpoB* and *rpsL*, conferring resistance to nalidixic acid, rifampicin and streptomycin, respectively. The plasmids were isolated from nature and the resistance mutations are polymorphic in natural populations of different species of bacteria [13]. These genes are involved in different steps of the cell's essential flow of information from DNA to protein. Specifically, *gyrA* codes for DNA gyrase, an enzyme involved in DNA replication. Nalidixic acid and other quinolones inhibit DNA replication by binding to DNA gyrase and resistance to this class of drugs arises from the prevention of this binding. Rifampicin belongs to the rifamycin class of antibiotics which bind to the β-subunit of RNA polymerase, coded by *rpoB*, thereby inhibiting transcription. Mutations in *rpsL*, which codes for ribosomal protein S12, interfere with translation and can produce resistance to streptomycin by blocking the binding of this drug to the ribosome 30S subunit. Secondly, using the same plasmids, we estimate epistasis between pairwise combinations of conjugative plasmids inside the same cell.

We find pervasive sign epistasis in the interaction between resistance mutations and conjugative plasmids. This implies that the acquisition of an additional resistance plasmid to the existing chromosomal resistance or the appearance of a chromosomal drug-resistance mutation in a bacterial cell already containing a plasmid may ameliorate the initial fitness cost of resistance and therefore complicate resistance reversal. We also observed an overall positive level of epistasis between mutations and plasmids. Both the chromosomal allele and the plasmid seem to contribute to determine the nature of the epistatic interaction, although the host genotype appears to have a more determinant effect. In contrast, the interaction between plasmids exhibit sign epistasis only once, and, despite the occurrence of several cases of somewhat strong epistasis, on average it appears to be null.

## Results

### Interaction between antibiotic resistance mutations and resistance plasmids

Pairwise epistasis, 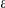, between loci A and B can be measured as follows. Suppose that the wild-type strain contains alleles A and B. If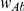 and 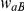 are the fitnesses of each of the single mutants relative to the wild-type strain, and 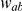 the relative fitness of the double mutant, then multiplicative epistasis is given by: 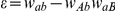. To estimate epistasis between plasmids and mutations, we defined these quantities in a similar way. If 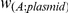 is the relative fitness of the strain with the wild-type allele (*A*) and containing a plasmid, 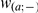
is the relative fitness of the mutant strain (with *A* allele replaced by the *a* allele), and 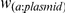 is the relative fitness of the strain containing both the mutation (*a* allele) and the plasmid, then epistasis between a plasmid and a chromosomal mutation becomes: 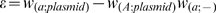
_._

Each conjugative plasmid was introduced in *E. coli* K12 MG1655 cells by conjugation. Then, we determined the fitness cost due to the presence of each plasmid relative to plasmid-free *E. coli* K12 MG1655 cells. This was performed using a competition assay, in the absence of antibiotics (see Materials and Methods). Fitness costs of plasmids span from 2.8% to 8% (Table S1).

The fitness cost imposed by ten different spontaneous antibiotic-resistance mutations was previously determined in ref. [27]. Table S2 presents the clones chosen from ref. [27] and the fitness costs of these mutations. Fitness costs of mutations vary between 0.5% and 27.5% (Table S2) [27].

To screen for epistatic interactions between chromosomal mutations and conjugative plasmids, we further constructed, by conjugation, all possible 50 combinations between these ten mutations and the five plasmids. Then we determined the fitness for each of these 50 combinations.

**Figure 1:**
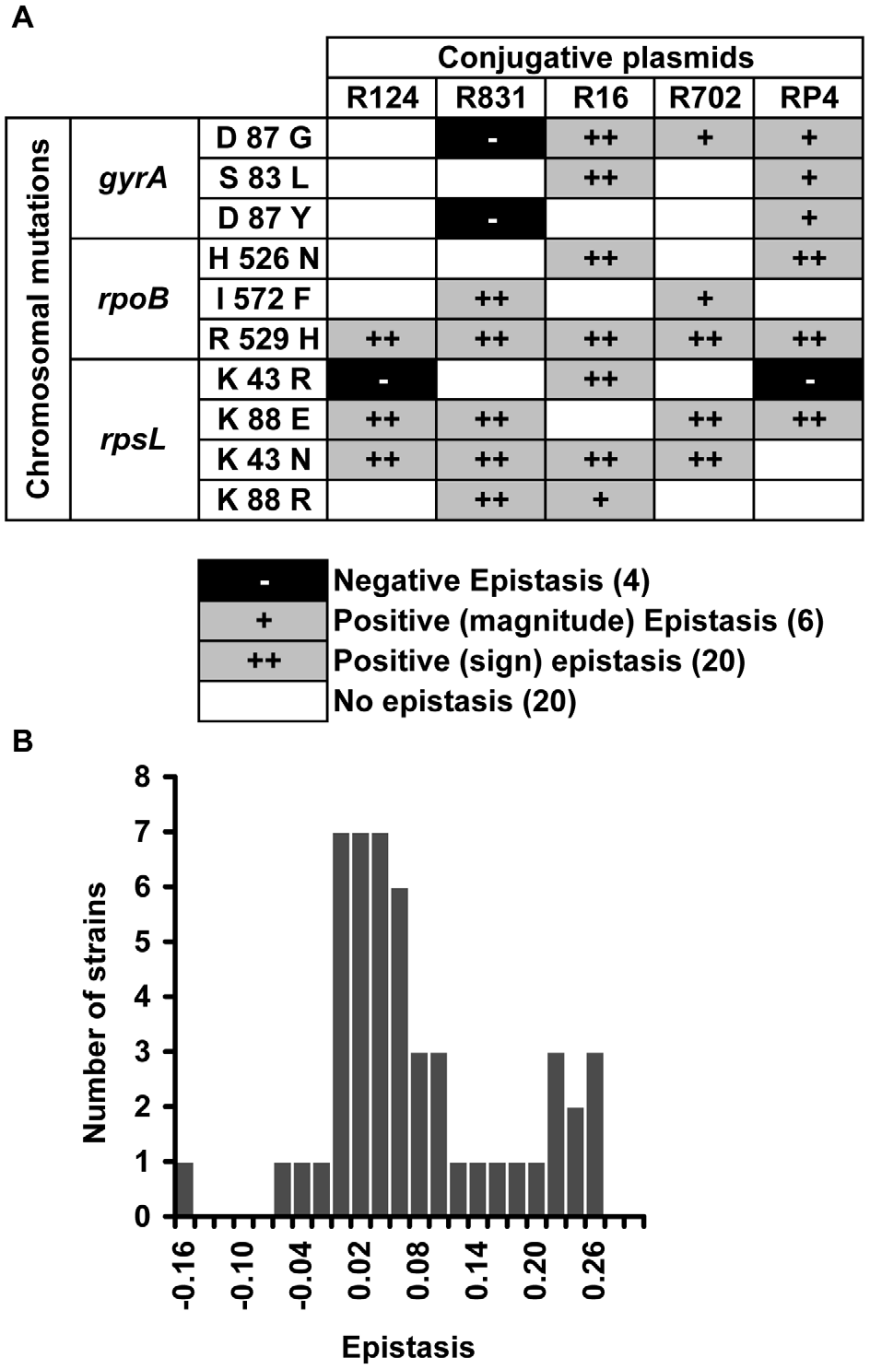
Epistasis between antibiotic resistance mutations and conjugative plasmids. (A) Epistasis between mutations in genes *gyrA*, *rpoB* and *rpsL* and conjugative resistance plasmids (positive epistasis in grey, negative in black and no epistasis in white). Sign epistasis is indicated with “++”. (B) Distribution of the epistasis values. Median is positive (0.037) with bootstrap 95% confidence interval [0.021; 0.065], showing an overall level of positive epistasis between chromosomal resistance mutations and conjugative resistance plasmids. According to a Shapiro-Wilk W-test, the ε values do not follow a normal distribution (p = 0.000968).

We found that 52% of the interactions present positive epistasis and only 8% present negative epistasis (Figure 1A). Figure 1A additionally shows that the nature of the epistatic interaction is not gene but allele specific. In fact, the conjugative plasmid influences how a specific allele interacts with plasmid-borne resistance determinants. This means that, depending on the plasmid, an allele can display no epistasis, positive epistasis or negative epistasis. For example, allele *gyrA D87G* exhibits no epistatic interaction with plasmid R124, however the same allele displays negative epistasis with plasmid R831 and positive epistasis with plasmids R16, R702 and RP4 (Figure 1A). The same pattern (allele specific nature of epistasis) had been observed for epistasis between resistance chromosomal mutations [27]. Supporting the pervasive nature of antagonistic interactions between mutations and plasmids, the distribution of the ε values (Figure 1B) has a significant positive median (median = 0.037, bootstrap 95% CI [0.021; 0.065]). Figure S1 plots the observed fitness against the fitness expected in the absence of epistasis (ε = 0).

To rule out the existence of compensatory mutations, we reconstructed five (double) combinations independently and in the opposite direction from what we did before: *gyrA* S83L(R16), *rpoB* I572F(R831), *rpoB* H526N(R16), *rpoB* R529H(R702), and *rpsL* K43N(RP4). We constructed these five clones by transducting [27] the antibiotic resistance mutation from our mutant *E. coli* strains into the wild-type strain (*E. coli* K12 MG1655) already containing the plasmid. Using this method we decreased the number of generations involving the antibiotic-resistance mutation by a half (because the plasmid was already there). In this way, we decrease the probability of occurrence of compensatory mutations. We measured the fitness of two independent clones corresponding to each of the five (double) combinations. For all five combinations, fitness values are not significantly different from the ones obtained using the previous method (Kruskal-Wallis, p>0.05). Four (*gyrA* S83L(R16), *rpoB* I572F(R831), *rpoB* H526N(R16) and *rpoB* R529H(R702)) of these five combinations correspond therefore to cases of sign epistasis (Figure 1A). One combination (*rpsL* K43N(RP4)) shows no interaction with these new independent clones as observed before (Figure 1A). The fact that independent clones exhibit the same fitness (and the same type of epistasis) shows that our results are robust. To further strengthen this point, we constructed two new independent clones of *rpoB* R529H(R702), this time using yet a different method: by simultaneous conjugation and transduction. The fitnesses of these clones were again not significantly different from before (Kruskal-Wallis, p>0.05).

**Figure 2:**
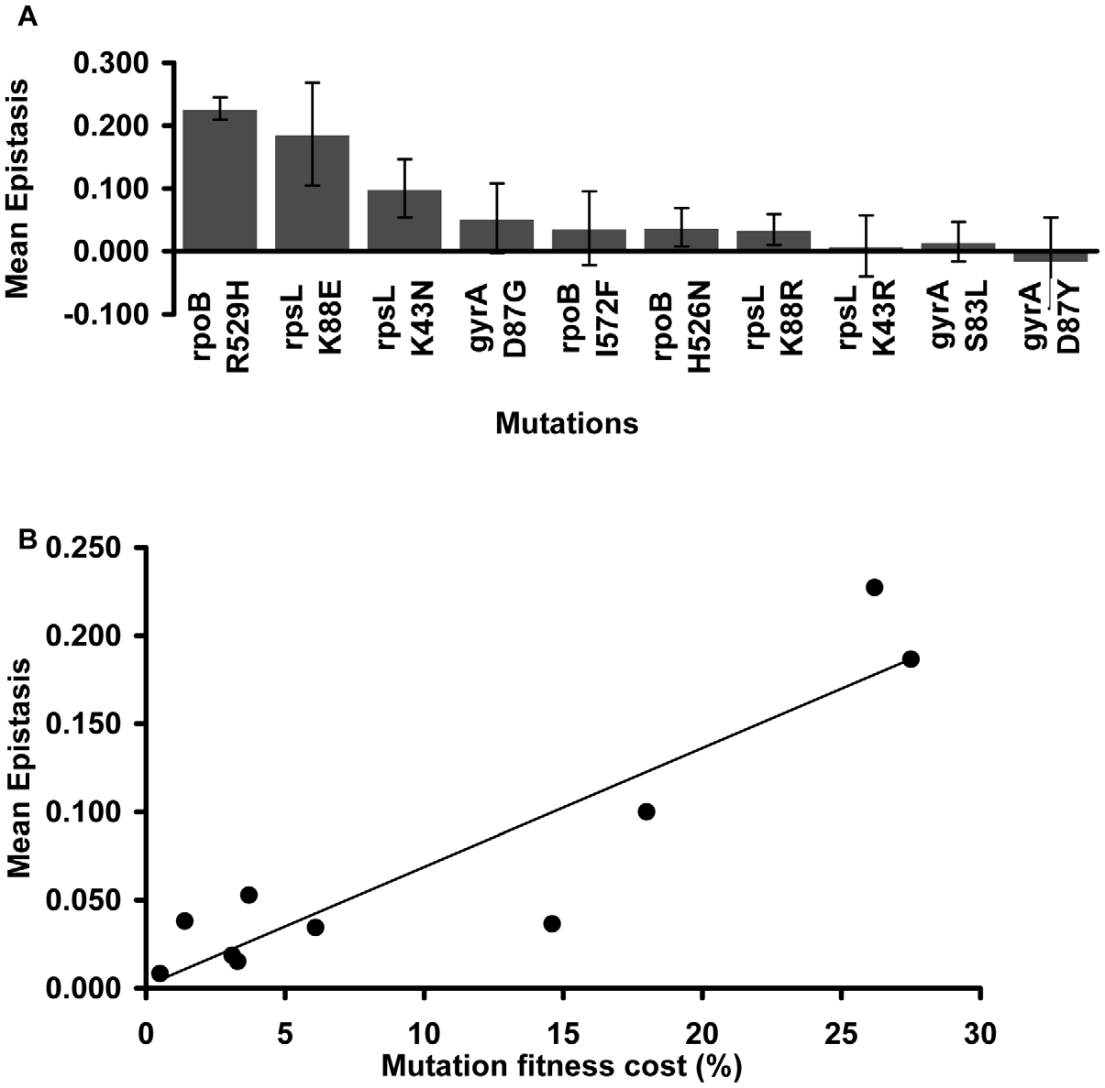
Mutation effect on the mean epistatic value. (A) Mean epistatic value for each mutation. Error bars indicate twice the standard error. Note how the mean epistatic effect significantly differ between mutations (Kruskal-Wallis p = 0.0016) (B) Mutations become increasingly epistatic as their severity increases. Note how the mean absolute epistatic effect correlates with the fitness cost associated with each mutation (Spearman p = 0.006).

Focusing on the resistance mutations, we notice that the mean epistatic value significantly varies among them (Kruskal-Wallis p = 0.0016). Figure 2A shows that mutations *rpoB* R529H, *rpsL* K88E and *rpsL* K43N exhibit positive and large ε values. The other mutations show lower mean ε values (Mann-Whitney U-Test, p = 0.000002).

Figure 2B shows that there is a significant correlation between the fitness cost created by a mutation and its mean epistatic value (deviation from zero in absolute value) (Spearman p = 0.006). In other words, mutations with a more deleterious effect on the cell tend to be more epistatic. This relationship had been initially proposed after *in silico* studies of digital organisms and theoretical modeling of RNA secondary structures [39]. Our results are in accordance with previous experimental data from studies of epistasis amongst antibiotic resistance alleles in *E. coli* [27], and from a study of enzymes involved in gene expression and protein synthesis in *Pseudomonas aeruginosa* [40].

**Figure 3:**
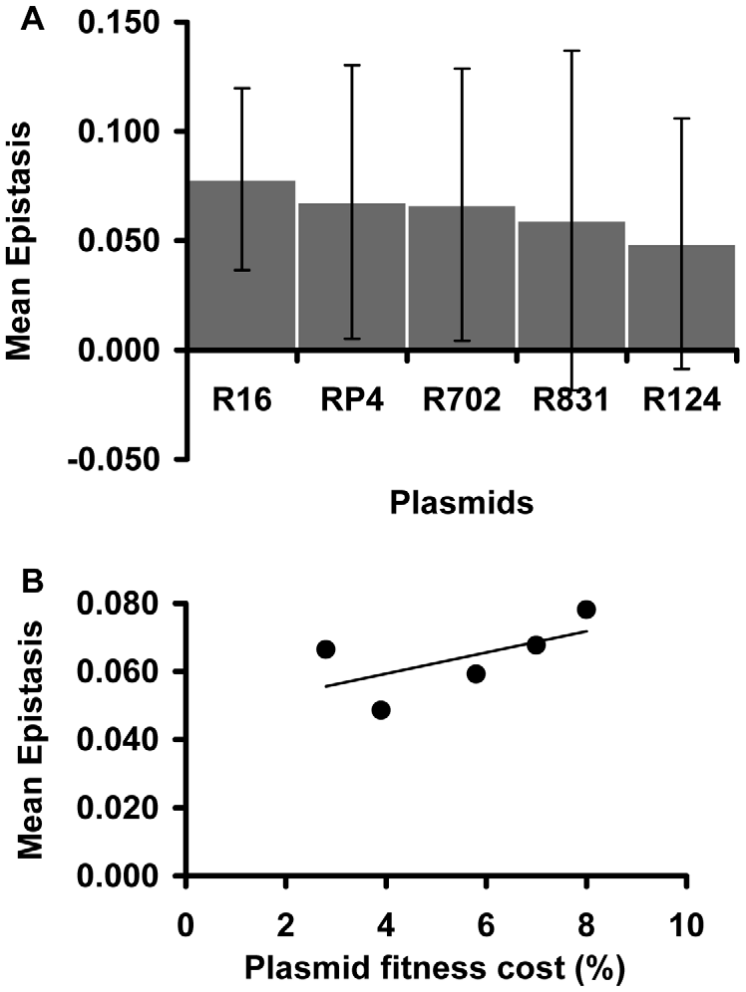
Plasmid effect on the mean epistatic value. (A) Mean epistatic value for each plasmid. Error bars indicate twice the standard error. Note how the mean epistatic effect does not significantly differ between plasmids (Kruskal-Wallis p = 0.6758). (B) Evidence for the lack of correlation between the fitness cost associated with each plasmid and its mean epistatic value (Spearman p = 0.188).

Focusing on the conjugative plasmids, Figure 3A shows their mean ε values. There are no significant differences in the mean ε values between plasmids (Kruskal-Wallis p = 0.676). This means that, on average, all studied conjugative plasmids tend to interact in the same way with chromosomal mutations. Moreover, comparison between Figure 2A and Figure 3A seems to suggest that the mutation (and not the plasmid) may be the major factor determining the type and the strength of the epistatic interactions we observed. In contrast to what was observed with the effect of mutations on epistasis [27], there is no significant correlation between the fitness cost created by a plasmid and its mean epistatic value (Figure 3B – Spearman p = 0.188). This may simply be due to lack of power, as variation in plasmids cost is much smaller than for mutations.

### Sign epistasis

**Figure 4:**
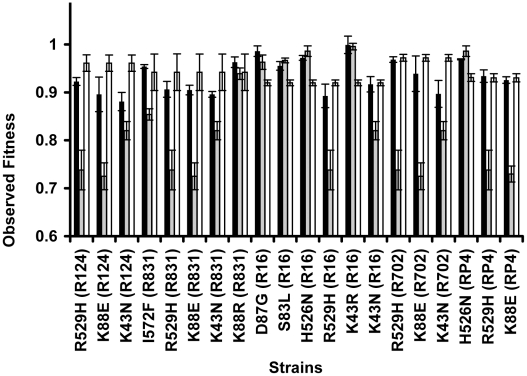
Sign epistasis between chromosomal mutations and conjugative plasmids. Sign epistasis occurs when the fitness of the strain carrying both resistance determinants (black bars) is greater than the fitness of at least one of the strains carrying a single resistance determinant (mutation – grey bars; or plasmid – white bars). The genotype D87G(R16) presents reciprocal sign epistasis [41]. Error bars represent twice the standard error.

A specific mutation can be deleterious on a particular genetic background and beneficial on others – a phenomenon known as sign epistasis. Strikingly, we report that 40% of the combinations between resistance chromosomal mutations and conjugative plasmids present sign epistasis (Figure 1A, where “++” indicates sign epistasis). These are cases where the strain carrying both resistant determinants was fitter than the strain carrying only the mutation or only the plasmid (Figure 4). One of the genotypes (D87G(R16)) presents reciprocal sign epistasis [41], meaning that the mutant D87G and harboring the R16 plasmid is fitter than both the plasmid-free mutant and the plasmid-bearing strain without the mutation (hence sensitive to nalidixic acid).

We found examples of sign epistasis in all resistance alleles and all plasmids of this study, i.e. there was no plasmid nor mutation where we did not find, at least one case of sign epistasis. This high prevalence of positive epistasis is not a consequence of plasmid transfer to the reference strain. The proportion of transconjugants was monitored at stationary phase, when bacterial density is higher than 10^9^ cells per ml, and was found to be less than 3% (Table S3). Also, computer simulations show that these plasmid transfer events imply an error in the calculation of epistasis that is less than 1%, hence less than the experimental error.

Another interesting aspect of our data is that, three out of the 50 combinations (plasmid+mutation) presented fitness costs not significantly different from zero (t-test, p>0.05); these strains are the following: *rpsL* K43R(R16), *rpsL* K43R(R831) and *gyrA* D87G(R16).

### Interaction between conjugative resistance plasmids

Finally, we measured epistasis between conjugative plasmids. This is relevant because there have been several reports of bacterial pathogens harboring multiple resistance plasmids [42], [43]. We constructed nine out of the 10 possible pairwise combinations of the five plasmids (plasmids R702 and RP4 belong to the same incompatibility group, thereby preventing the construction of this double transconjugant). In this context, epistasis was estimated as: 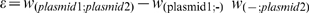
_._

In this mathematical expression, 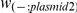 and 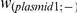 are the fitnesses of single-plasmid carrying strains relative to the wild-type plasmid-free strain, and 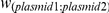 is the fitness of the strain carrying both plasmids, relative to the same wild-type plasmid-free strain.

**Figure 5:**
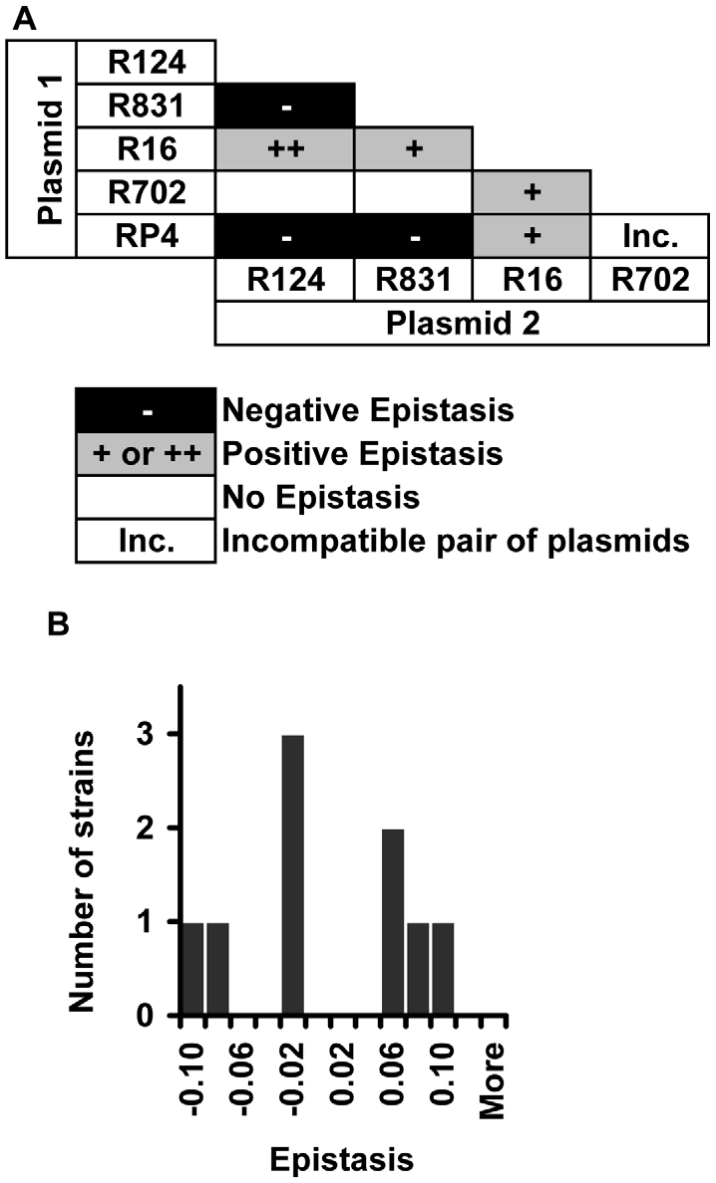
Evidence for epistasis between conjugative plasmids. (A) Distribution of the types of epistatic interaction found between conjugative plasmids (positive epistasis in grey, negative epistasis in black and no epistasis in white). “Inc” indicates a combination of incompatible plasmids. The single case of sign epistasis is marked with ‘++’. (B) Distribution of the epistasis level, ε, whose median is −0.00083 with bootstrap confidence interval [−0.035; 0.061], showing that there are several cases of strong positive and negative epistasis despite the near 0 median.

Two different plasmids inside the same bacterial cell can interact either antagonistically or synergistically (Figure 5A). Epistatic interaction was found in 7 out of 9 (78%) strains. Positive epistasis (antagonistic interaction) is nearly as frequent (4/9) as negative epistasis (3/9). One out of nine pairwise combinations of plasmids presented sign epistasis (“++” in Figure 5A). Figure 5B shows the distribution of ε values for all pairwise combinations of plasmids. On average, plasmid pairwise epistasis is close to 0 (median  = −0.000830, bootstrap 95% CI [−0.034690; 0.061360]). It is interesting to note that one of the plasmids, R16, interacts antagonistically with all other plasmids, and also with most mutations. This plasmid is also the most costly (Table S1).

## Discussion

Our results show that 52% (26/50) of the combinations between antibiotic resistance mutations and resistance conjugative plasmids interact antagonistically. This is a remarkable result because the fitness cost of these strains that carry both resistance determinants is lower than the independent sum of the cost of each determinant. Moreover, 20 out of these 26 antagonistic interactions (77%) exhibit sign epistasis or reciprocal sign epistasis, also an outstanding finding because it means that the fitness cost of harboring both resistance determinants is lower than the fitness cost of bearing one of them. In other words, an initially deleterious antibiotic resistance mutation can become beneficial through the acquisition of a transferable antibiotic resistant plasmid (16 cases); likewise, an initially costly antibiotic resistant plasmid may become beneficial through the acquisition of a mutation conferring resistance to an additional antibiotic (5 cases). This adds up to 20 cases of sign epistasis because of one instance of reciprocal sign epistasis. Last, but not least, three of the plasmid+mutation combinations presented fitnesses not significantly different from the fitness of the wild-type strain.

Positive epistasis has been shown to occur between resistance alleles in multidrug resistant *E. coli*. [27], *P. aeruginosa* [44] and *Streptococcus pneumoniae* [45]. Such phenomena reduce the fitness cost associated with multidrug resistance and may drive its spread. Our study aimed to detect the putative occurrence of epistatic interactions involving conjugative resistance plasmids. Such knowledge may help predict how a bacterial population will evolve after the introduction of plasmid-borne resistance determinants through horizontal gene transfer.

Our data strikingly suggests the pervasive occurrence of sign epistasis in the interaction between chromosomal antibiotic resistance mutations and conjugative plasmids. Sign epistasis has been shown to have the power to constrain protein adaptation by limiting the number of possible mutational paths and is therefore relevant to the understanding of multidrug resistance emergence [46]. Moreover, bacterial adaptation to the cost of mutation-determined resistance involves the acquisition of second-site mutations that compensate the fitness cost of the original mutation [10]. Thus, compensatory mutations are an example of sign epistasis [3], [10], [11], [46]. Our finding of pervasive sign epistasis with conjugative plasmids is one of the worst possible scenarios for the current efforts to eradicate resistance through antibiotic bans. Sign epistasis allows strains carrying a resistance mutation and a plasmid to exhibit higher fitness, thus being able of outcompeting strains carrying only the mutation or the plasmid (depending on the specific case). These results pinpoint the need for future studies involving other plasmids and other resistances.

Also important in the context of antibiotic resistance is our finding of the ubiquitous occurrence of positive epistasis between resistance plasmids and chromosomal resistance mutations. If such antagonistic interaction is a common phenomenon, then multidrug resistance determined by the simultaneous presence of plasmid-borne and chromosomal determinants will not create such a high fitness cost as one could predict based on the individual costs. Hence, such multiresistant strains may be able to persist at significant frequencies in populations where the antibiotic selective pressure has been removed.

Our findings are in accordance with the results of a large-scale survey for genes of the *E. coli* chromosome that are affected by the presence of the conjugative F-plasmid [47]. Such study found 107 genes exhibiting epistatic effects with the F-plasmid. Although such effect was not found for *gyrA*, *rpoB* and *rpsL*, other host genes involved in information transfer were reported to be affected by the presence of the F-plasmid [47]. Under the framework of the complexity hypothesis, these interactions between plasmids and informational genes (*rpoB* [48], [49], [50] and *rpsL* [51]) and a topoisomerase (*gyrA* [52]) are expected, given their pleiotropic interactions with other genes. For example, Schmitt et al. have shown that certain *rpoB*, *rpsL* and *gyrA* alleles affect F-exclusion of bacteriophage T7 [53]. In addition, Ozawa et al. [54] showed that *rpoB* mutations interact with a plasmidic gene (in *Enterococcus faecalis*). Similarly, *gyrB* may also interact with plasmids, eventually leading to their elimination from cells [55]. In conclusion, resistance genes present on plasmids are not necessarily responsible for the epistatic interactions observed.

We also report here the occurrence of significant epistasis between two types of conjugative plasmids within the same host. This finding has relevance for clinical isolates exhibiting multidrug resistance afforded by the co-existence of several plasmids, a situation which appears to be relatively common [43]. Our data indicates that, on average, epistasis between the conjugative plasmids is close to zero. However, we do not believe that our results suggest a tendency for no epistatic interactions between conjugative plasmids. In fact, our near-zero median level of epistasis between conjugative plasmids is the consequence of having a similar frequency of somewhat strong positive and negative epistatic interaction pairs. Our results may indicate that plasmid interaction follows an all-or-nothing type of response where the net epistatic effect is either strongly negative or strongly positive. However, further studies should use a larger sample of plasmids. Recently, *in silico* studies of *E. coli* and *S. cerevisiae* metabolic networks have suggested that genes involved in essential reactions tend to interact antagonistically, while negative epistasis was mainly limited to non-essential gene pairs [56]. The accessory nature of plasmids versus the essential role of *gyrA*, *rpoB* and *rpsL* in information flow may explain why positive epistasis appears to be more frequent in the interaction between chromosomal mutations and a plasmid than between two types of plasmids.

Our finding of pervasive positive epistasis and, in particular, of sign epistasis, between mutations and conjugative plasmids raises serious concerns to the reversal of antimicrobial-drug resistance. Plasmid-borne multidrug resistance is widespread in microbial clinical, animal and environmental isolates. Dissemination is facilitated by the conjugative plasmids' ability to mobilize their own transfer (and of other plasmids) from the original host to a new cell. Many plasmids are even able to move between phylogenetically distant organisms. Furthermore, it is known that plasmids act as recruiting platforms for resistance genetic determinants, many of them able to transpose between the plasmid and the host chromosome (and vice-versa). Thus, and given the widespread nature of horizontal gene transfer in prokaryotes it has been suggested that microbes share a common gene pool [57]. Therefore, we predict that plasmid-borne resistance dissemination control through antibiotic bans is not likely to be successful. We suggest that resistance reversal policies must target plasmids vulnerabilities. Three approaches have been suggested [58]: inhibition of plasmid conjugation, inhibition of plasmid replication, and exploitation of plasmid-encoded toxin-antitoxin systems.

## Materials and Methods

### Bacterial strains, plasmids, and growth conditions

We used five natural conjugative plasmids, R124, R702, R16, R831, and RP4, kindly provided by the Institute for Health, Environment and Safety of the Belgian Nuclear Research Centre. Plasmid characteristics are listed in Table S1. We introduced these plasmids in wild-type *E. coli* K12 MG1655 and in a set of 10 spontaneous antibiotic-resistant clones derived from the wild-type strain (Table S2). These mutations have been previously mapped to *gyrA*, *rpoB* and *rpsL* resulting in resistance to nalidixic acid, rifampicin and streptomycin (ref. 27).

For the construction of bacterial strains with conjugative plasmids, donors and recipients (either wild-type *E. coli* K12 MG1655 or strains shown in Table S2) were put together for 24 hours. All donor strains are auxotrophic for specific amino-acids and/or unable to use maltose, due to deletions in essential genes/operons, as indicated in chromosomal markers: Mal^−^: maltose; Trp^−^: tryptophan; Met^−^: methionine and Pro^−^: proline. Selection of transconjugants was performed in M9 minimal medium (56.4 g/L M9 minimal salts, 2 mM magnesium sulfate, 4 g/l sugar (see bellow), 15 g/l agar), supplemented with the appropriate antibiotics. If donors are auxotrophic (Table S4) for two amino-acids (*E. coli* CM140 and *E. coli* CM597), transconjugants were selected on minimal medium plates containing glucose and no amino-acids. Otherwise, we used maltose and tryptophan (*E. coli* CM317, *E. coli* CM319, *E. coli* CM312). As a control we confirmed that neither donors (due to auxotrophies or inability to use maltose as carbon source) nor recipient (due to antibiotics selecting for plasmidic resistance genes) grow on these plates.

Transduction was done with P1 bacteriophage, according to the methods described by Trindade et al. [27].

In competition assays, we used *E. coli* K12 MG1655 Δara as “reference strain”. Due to a deletion in the arabinose operon this strain produces red colonies when grown in tetrazolium arabinose (TA) indicator agar, allowing it to be distinguished from its competitor, which produces white colonies. TA medium contains 1% peptone, 0.1% yeast extract, 0.5% sodium chloride, 1.5% agar, 1% arabinose and 0.005% tetrazolium chloride.

All bacterial strains were grown in liquid Luria-Bertani (LB) medium at 37°C with agitation. Solid media was obtained by the addition of agar (15 g/l). For growth and transconjugant selection, antibiotics were added as follows: 40 µg/ml of nalidixic acid, 100 µg/ml of rifampicin, 100 µg/ml of streptomycin, 20 µg/ml of tetracycline, 100 µg/ml of kanamycin and 100 µg/ml of ampicillin.

Dilutions of cultures were done in MgSO_4_ 0.01 M. All strains were kept frozen in 15% glycerol stocks.

### Fitness assays

Competition assays were performed to determine the fitness cost of the resistance determinants, either the plasmid carriage alone, the coexistence of both plasmid and mutation or the carriage of two plasmids. The method used has been previously described by ref. [27]. The strains carrying resistance determinants were competed against a susceptible reference strain, *E. coli* K12 MG1655 Δara, in an approximate proportion of 1∶1 and in the absence of antibiotic selective pressure. (i) Both strains were grown in 10 ml of liquid LB medium for 24 hours at 37°C with aeration. (ii) 50 µl of the dilution 10^−4^ of each strain was added to 50 ml screw-cap tubes containing 10 ml of liquid LB medium. (iii) Values of both strain's initial ratio were estimated by plating a dilution of the mixture in TA agar medium. (iv) Competitions proceeded by a period of 24 hours at 37°C with aeration. (v) At the end of the competition, appropriate dilutions were plated onto TA agar plates to obtain the final ratios of both competitors. These competitions spanned about 19 to 22 bacterial generations. If a high fitness cost precluded the resistant strain of being recovered in the TA plates, a smaller dilution was plated onto minimal medium supplemented with arabinose, which does not allow the growth of the reference strain. The fitness cost of each strain – i.e. the selection coefficient, s, – was estimated as the per generation difference in Malthusian parameters between the mutant and the wild-type (r_m_ and r_w_ respectively): specifically, 
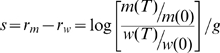
, where T is the final time and g is the total number of generations from t = 0 until t = T. Then, we discounted the cost of the Δara marker [59]. The fitness cost was estimated as an average of three independent competition assays.

### Measurement of epistasis and statistical significance

As explained in the main text, epistasis between a mutation and plasmid can be calculated as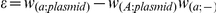, where 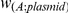 is the relative fitness of the strain with the wild-type allele (*A*) and carrying the plasmid, 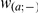
is the relative fitness of the mutant strain (with *A* allele replaced by the *a* allele), and 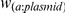 is the relative fitness of the strain containing both the mutation (*a* allele) and the plasmid. Similarly, we defined epistasis between plasmids as 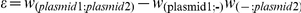, where 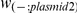 and 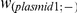 are the fitnesses of single-plasmid strains relative to the wild-type plasmid-free strain, and 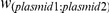 is the fitness of the strain carrying two types of plasmids, relative to the same wild-type plasmid-free strain. Then, the error (σ_ε_) of the value of ε is estimated by the method of error propagation;

for pairwise combinations of mutation and plasmid:



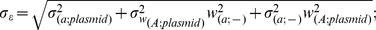
for pairwise combinations of plasmids:



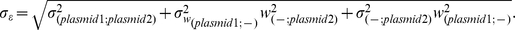



If the value of ε was within the calculated error, we considered that the two resistance determinants (mutation and plasmid or plasmid and plasmid) did not show significant epistasis (indicated as white boxes labeled “no epistasis” in Figure 1A and Figure 5A). From the distribution of values of ε, provided in Figure 1B and Figure 5B, we calculated the median value of ε and its 95% CI by bootstrap where we took 10 000 samples.

To test the presence of sign epistasis, we compared the fitness of each strain carrying two resistance determinants (mutation and plasmid or plasmid and plasmid) and its corresponding single resistance-determinant strains. We used a Student t-test to assess if the fitness of the double-resistance-determinants strain was higher than the fitness of any of the single resistance-determinant strains.

Statistical analyses performed using software Statistica 9.0 and MatLab R2009b. Computer simulations performed with Mathematica 7.

## Supporting Information

Figure S1 Evidence for positive epistasis between plasmids and mutations. Relation between the observed fitness of the strains carrying a resistance mutation and a conjugative plasmid and the expected fitness under the assumption of no epistasis (represented by the line). Most points (52%) are significantly above the line. Error bars represent twice the standard error.
(TIF)

Table S1 List of the conjugative plasmids used in the present study.
(DOC)

Table S2 List of antibiotic resistant mutants and fitness cost.
(DOC)

Table S3 Monitoring of conjugative transfer of plasmids to reference strain.
(DOC)

Table S4 List of plasmid donors used for strains construction.
(DOC)
