# Supplementary figures and images for: Pervasive Sign Epistasis between Conjugative Plasmids and Drug-Resistance Chromosomal Mutations
Source: PLoS Genet. 2011 Jul 28;7(7):e1002181. doi: 10.1371/journal.pgen.1002181 (PMC3145620; doi:10.1371/journal.pgen.1002181)

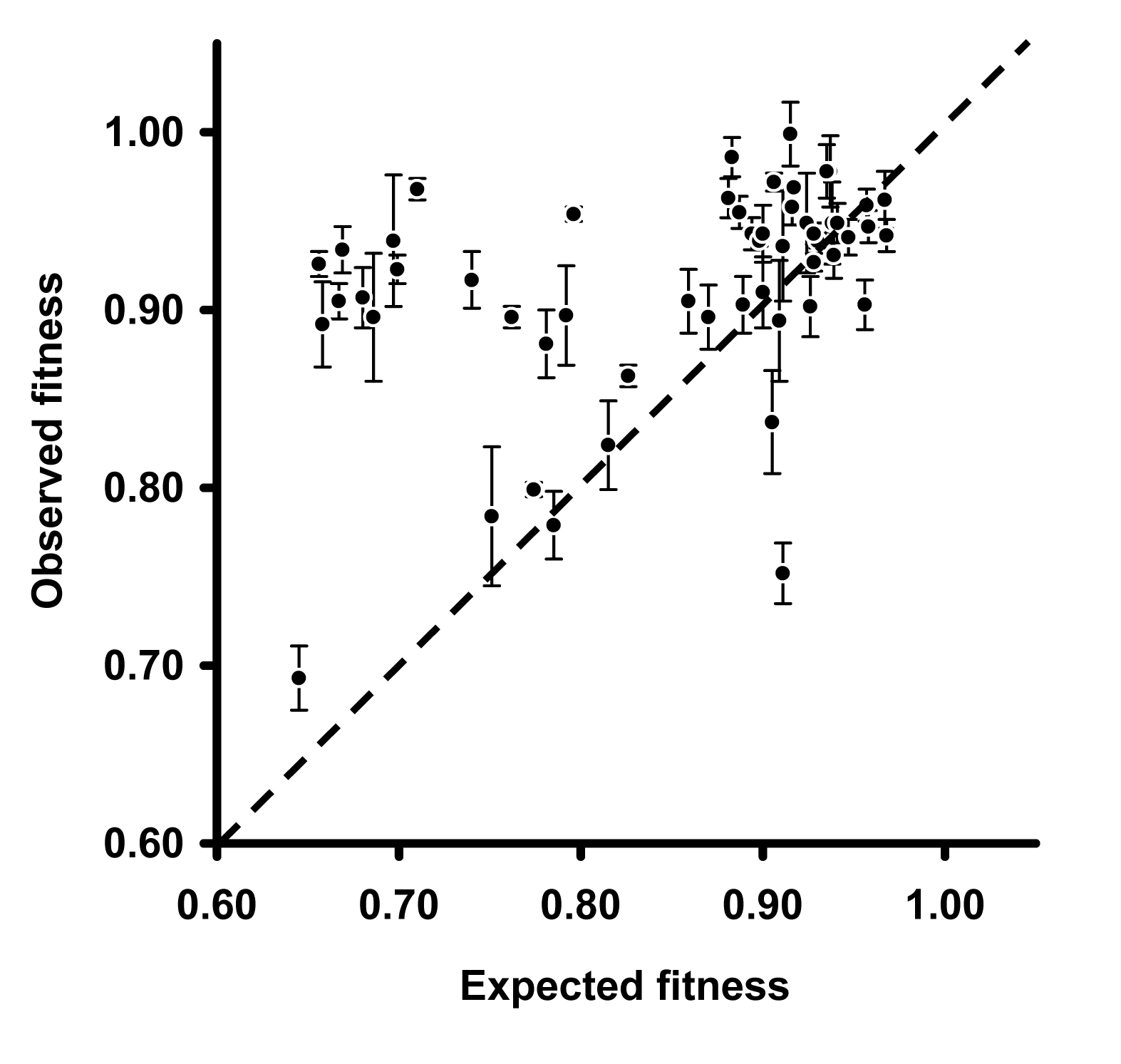

Supplement: Figure S1 — Evidence for positive epistasis between plasmids and mutations. Relation between the observed fitness of the strains carrying a resistance mutation and a conjugative plasmid and the expected fitness under the assumption of no epistasis (represented by the line). Most points (52%) are significantly above the line. Error bars represent twice the standard error. (TIF) [file pgen.1002181.s001.tif]
