# Supplementary material for: Pervasive Sign Epistasis between Conjugative Plasmids and Drug-Resistance Chromosomal Mutations
Source: PLoS Genet. 2011 Jul 28;7(7):e1002181. doi: 10.1371/journal.pgen.1002181 (PMC3145620; doi:10.1371/journal.pgen.1002181)
Supplement: Table S1 — List of the conjugative plasmids used in the present study. (DOC) [file pgen.1002181.s002.doc]

Table S1. List of the conjugative plasmids used in the present study. We present their incompatibly group, antibiotic resistance markers, size, the fitness cost they create when inserted in *Escherichia coli* K12 MG1655, and origin.

| **Plasmid** | **Incompatibility group** | **Antibiotic Resistance Markers*** | **Size**  **(kb)** | **Fitness Cost (%) (2*standard error)** | **Origin of plasmid** | **References** |
| --- | --- | --- | --- | --- | --- | --- |
| **R124** | IncF IV | Tc | 125.7 | 3.9 (1.7) | *Salmonella enterica* serovar Typhimurium | [1,2] |
| **R831** | IncL | Km, Sm | ? | 5.8 (3.8) | *Serratia marcescens* | [3] |
| **R16** | IncB | Ap, Sm, Su, Tc | 104 | 8.0 (0.6) | *E.coli* | [4] |
| **R702** | IncP | Km, Sm, Su, Tc | 69.7 | 2.8 (0.7) | *Proteus mirabilis* | [5] |
| **RP4** | IncP1 | Ap, Km, Tc | 60.1 | 7.0 (0.9) | *Pseudomonas aeruginosa* | [6*,*7] |

*Ap: ampicillin; Km: kanamycin; Sm: streptomycin; Su: sulphonamides; Tc: tetracycline.

1. Hedges RW, Datta N (1972) R124, an fi R factor of a new compatibility class. Journal of General Microbiology 71: 403-5.

2. Youell J, Firman K (2008) EcoR124I: from plasmid-encoded restriction-modification system to nanodevice. Microbiology and Molecular Biology Reviews 72: 365-77.

3. Hedges RW, Rodriguez-Lemoine V, Datta N (1975) R factors from *Serratia marcescens*. Journal of General Microbiology 86: 88-92.

4. Hedges RW, Datta N, Kontomichalou P, Smith JT (1974) Molecular specificities of R factor-determined beta-lactamases: correlation with plasmid compatibility. Journal of Bacteriology 117: 56-62.

5. Hedges RW, Jacob AE (1974) Transposition of ampicillin resistance from RP4 to other replicons. Molecular & General Genetics 132: 31-40.

6. Datta N, Hedges RW, Shaw EJ, Sykes RB, Richmond MH (1971) Properties of an R factor from *Pseudomonas aeruginosa*. Journal of Bacteriology 108: 1244-9.

7. Pansegrau W, Lanka E, Barth PT, Figurski DH, Guiney DG, et al. (1994) Complete nucleotide sequence of Birmingham IncP alpha plasmids. Compilation and comparative analysis. Journal of Molecular Biology 239: 623-63.
