# Supplementary material for: Pervasive Sign Epistasis between Conjugative Plasmids and Drug-Resistance Chromosomal Mutations
Source: PLoS Genet. 2011 Jul 28;7(7):e1002181. doi: 10.1371/journal.pgen.1002181 (PMC3145620; doi:10.1371/journal.pgen.1002181)
Supplement: Table S2 — List of antibiotic resistant mutants and fitness cost. (DOC) [file pgen.1002181.s003.doc]

Table S2. List of antibiotic resistant mutants and fitness cost.

| **Gene** | **Genotype: amino acid change; nucleotide change^†^** | **Cost (2*standard error) %^†^** | **Resistance^¶^** |
| --- | --- | --- | --- |
| *gyrA* | D 87 G ; GAC to GGC | 3.7 (1.5) | Nal |
|  | S 83 L; TCG to TTG | 3.3 (1.2) | Nal |
|  | D 87 Y; GAC to TAC | 3.1 (1.9) | Nal |
| *rpoB* | H 526 N; CAC to AAC | 1.4 (1.1) | Rif |
|  | I 572 F; ATC to TTC | 14.6 (1.2) | Rif |
|  | R 529 H; CGT to CAT | 26.2 (4.9) | Rif |
| *rpsL* | K 43 R; AAA to AGA | 0.5 (1.4) | Sm |
|  | K 88 E; AAA to GAA | 27.5 (2.8) | Sm |
|  | K 43 N; AAA to AAC | 18.0 (1.9) | Sm |
|  | K 88 R; AAA to AGA | 6.1 (1.2) | Sm |

**¶** Nal: nalidixic acid; Rif: rifampicin; Sm: streptomycin

† Data from ref. 27 (main text).
