# Supplementary material for: Pervasive Sign Epistasis between Conjugative Plasmids and Drug-Resistance Chromosomal Mutations
Source: PLoS Genet. 2011 Jul 28;7(7):e1002181. doi: 10.1371/journal.pgen.1002181 (PMC3145620; doi:10.1371/journal.pgen.1002181)
Supplement: Table S3 — Monitoring of conjugative transfer of plasmids to reference strain. (DOC) [file pgen.1002181.s004.doc]

Table S3**.** Monitoring of conjugative transfer of plasmids to reference strain.

|  | Total cell density (cfu/mL)^*^ | Transconjugants (% of Δara)^#^ |
| --- | --- | --- |
| **R124** | (1.29± 0.86) x 10^9^ | 1 |
| **R702** | (1.03± 0.09) x 10^9^ | 2 |
| **R16** | (1.08± 0.12) x 10^9^ | <0.5 |
| **R831** | (1.17± 0.06) x 10^9^ | <0.5 |
| **RP4** | (1.30 ± 0.17) x 10^9^ | 1 |

* Colony forming units per milliliter; # percentage of recipients that received the plasmid (transconjugants) at stationary phase.
