# Supplementary material for: Pervasive Sign Epistasis between Conjugative Plasmids and Drug-Resistance Chromosomal Mutations
Source: PLoS Genet. 2011 Jul 28;7(7):e1002181. doi: 10.1371/journal.pgen.1002181 (PMC3145620; doi:10.1371/journal.pgen.1002181)
Supplement: Table S4 — List of plasmid donors used for strains construction. (DOC) [file pgen.1002181.s005.doc]

Table S4. List of the plasmid donors used for strain construction.

| Bacterial strain | Plasmid harbored | Chromosomal markers |
| --- | --- | --- |
| *E. coli* CM317 | R124 | Mal^-^ Trp^-^ |
| *E. coli* CM319 | R831 | Mal^-^ Trp^-^ |
| *E. coli* CM312 | R16 | Mal^-^ Trp^-^ |
| *E. coli* CM597 | R702 | Met^-^ Pro^-^ |
| *E. coli* CM140 | RP4 | Met^-^ Pro^-^ |
